# Placebo Effects in Neurological Diseases

**Published:** 2010-05-25

**Authors:** A Dumitriu, BO Popescu

**Affiliations:** * Department of Neurology, University Hospital Bucharest, ‘Carol Davila’ University of Medicine and PharmacyRomania; ** Laboratory of Molecular Medicine, ‘Victor Babes’ National Institute of Pathology, BucharestRomania

**Keywords:** classical conditioning, Parkinson's disease, neuropathic pain, headache, multiple sclerosis, epilepsy, randomized controlled trials, ethical aspects

## Abstract

There is an imperious need of redefining placebo effect in contemporary times. The effects of sham medical intervention, combined with a careful observation of the natural evolution of a disease, could reveal the true efficiency and impact of active drugs. This interest is not driven only by a scientific curiosity, but also by the pragmatic fact that the standard process of approving new medicines through supportive clinical trials requires a comparison against placebo. A complete understanding of the placebo effect should include both its psychological mechanisms and the underlying neurobiology. In contrast to other type of conditions, neurological disorders could provide specific clues in understanding the placebo effect, since the pathogenic mechanisms of different diseases might interfere with neuronal circuitry involved in the perception of disease symptoms. However, there are ethical considerations dictating the limits of using placebo. This paper reviews recent articles about placebo effect, with an emphasis on its importance in several neurological conditions (Parkinson's disease, neuropathic pain, headache, multiple sclerosis, epilepsy), and intends to offer new insights on this major topic.

## Introduction

The term ‘placebo’, which comes from the Latin language and means ‘I shall please’, is medically used to designate a biologically inert substance or a sham surgical intervention that produces a ‘placebo effect’, which is defined as a favorable outcome in the course of a disease state. Some authors characterize placebo effect as a form of interpersonal healing, distinct from the natural evolution of a disease, which is present in some degree in all healing encounters [1,2]. The body of evidences on placebo effects is currently growing in an accelerated fashion, as there are annually thousands of publications presenting randomized placebo–controlled trials. The spectrum of favorable effects noticed when using placebo in neurological conditions includes the relief of various types of pain, such as headache and neuropathic pain, improvement of manifestations of Parkinson's disease, reduction of the seizure frequency in epilepsy, alleviation of symptoms in multiple sclerosis, as well as a fortunate impact on cognitive and mood disorders. 

The recent developments in brain imaging enabled a better understanding of the neurobiological mechanisms underlying the placebo effects, of which the opioid and dopaminergic systems are the most reliably documented. Placebo is the gold standard for the comparison of a new drug in clinical trials, but its effects cannot always be properly isolated by trial design, which is a matter of dispute in the scientific community. Ethical issues regarding the use of placebo are also a hot topic nowadays, and careful attention is recommended in order to minimize its effects in the clinical research; however, there are many voices that have a positive attitude toward using placebo in clinical settings. 

Different reviews of literature have been recently performed to investigate the placebo effects in the neurological disorders, the psychological aspects facilitating placebo, the evidences about its neurobiological basis, the current views about using placebo in clinical trials and the ethical questions raised by its use.

### Psychological aspects of placebo effects

Several psychological mechanisms seem to contribute to the appearance, enhancement or duration of the placebo effects namely classical conditioning and cognitive factors, such as expectation, desire and reward[3,4,5]. The most important remains the classical conditioning, which has been studied in research trials by administering an active drug before giving placebo or by reducing pain intensity of a stimulus at the same time a placebo is given. Conditioning is actually an ubiquitous psychological mechanism, as personal previous long–term experiences of a patient will influence the results: doctor–patient relationship, the trial site, the aspect of the pill, the route and the frequency the treatment is given [6]. 

The patient's expectation of clinical improvement is also responsible for the onset and tailoring of the placebo effects. The importance of expectancy has been revealed in studies with a balanced placebo design, which can be schematically described as the assignment of patients to one of four groups: active treatment group where they were told they are given active treatment, active treatment group where they were told they are given placebo, placebo group where they were told they are given active treatment, placebo group where they were told they are given placebo. One study with this type of design performed in cocaine abusers showed that, for the group that received methylphenidate, the brain glucose metabolism was higher in those who were told they were given methylphenidate than in those who were told they were given placebo [6].

It is also important that the patient has a sense of control over the disease by being actively involved in the treatment administration, as this will enhance the odds of a favorable effect. Moreover, higher placebo effects were elicited when patients were given a firm diagnosis, performed diagnostic tests, told that a novel procedure would be used and encouraged they would get better [6]. It is believed that the placebo effects can be enhanced by using verbal suggestions that increase the expectation of pain relief and decrease the perceived necessity of pain reduction; a careful avoidance of these suggestions can decrease the placebo effects [7]. One study showed that inducing strong expectation of analgesia before sleep could be a method of enhancing the placebo effects [8].

Another study found a significant correlation between some personality traits, like behavioral drive, novelty and fun seeking (which are together described as ‘dopamine related traits’), and the propensity to develop a placebo analgesic response [9].

The outcome of a disease is indeed influenced by the doctor–patient relationship. The doctor's attitude triggers some favorable or unfavorable neuropsychological mechanisms, which will lead to illness alleviation or aggravation, respectively. It is interesting to note that the patients have the tendency to report better placebo outcomes than the clinicians, as one study on facial skin rejuvenation revealed–here the improvements were perceived only by the subjects, but not by the doctors or the blinded experts [10].

### Neurobiological basis of placebo effects

The first important step in understanding the biological basis of the placebo effect was taken by Levine et al in 1978, who discovered that the analgesic effect obtained by using an inert substance was blocked by naloxone, an opioid antagonist. Later, a more detailed picture about the involvement of opioid system came into shape and further arguments were gathered. For instance, an enhancement of placebo–induced analgesia was obtained by using proglumide, an antagonist of cholecystokinin that acts on the opioid system [11].

Today a consensus was reached that the endogenous opioid system, by the activation of its µ–opioid receptors, is the main mediator of the placebo effects in various types of pain, as shown by the measurement of the blood flow by using functional magnetic resonance imaging (fMRI) and positron emission tomography (PET) with µ–opioid receptor selective radiotracer 11C carfentanil.During placebo–induced analgesia, a reduction in the activation of pain–sensitive brain areas, such as rostral anterior cingulate cortex, prefrontal cortex, insula, thalamus, amygdala, nucleus accumbens and periaqueductal gray matter was noted [12,13,14].  More detailed PET investigations of placebo analgesia found the following involved territories: rostral anterior cingulate, orbitofrontal and dorsolateral prefrontal cortex, anterior and posterior insula, nucleus accumbens, amygdala, thalamus, hypothalamus, periaqueductal grey matter, dorsal raphe and cuneiformis nuclei [15,16]. 

In one study, naloxone was used to decrease placebo analgesia by using fMRI. The CNS structures involved in this process were revealed as being rostral anterior cingulate cortex, periaqueductal gray matter, hypothalamus and rostral ventromedial medulla [17]. Placebo effect is mediated not only at the supra–spinal level, but also in the spinal cord [18]; this finding was documented in one study that used fMRI to bring direct evidence that, when placebo is used, pain–related activity is also decreased in the spinal cord [19]. However, the CNS activations induced by placebo analgesia and by opioids are only partially superposable, the amplitude of activation being higher for the opioid substances [6]. 

Placebo analgesia is mediated not only by the opioid system, but also by other pathways. It is thought that expectation uses the opioid system to induce placebo analgesia, but the conditioning may use different biochemical systems, according to the drug initially administered. When an opioid substance is used for conditioning, placebo analgesia occurs via opioid system, and can be blocked by naloxone; when another type of drug is used, for instance ketorolac, another system mediates placebo analgesia, and therefore analgesia cannot be antagonized by naloxone [20].

A dichotomy was established between the transitory and sustained analgesic effects of placebo, and it was shown, while using fMRI, that the effects with different temporal profiles have distinct CNS origins. The transitory component of placebo analgesia is managed by cognitive cerebral areas, like language centers in the dominant hemisphere and executive functioning centers in the non–dominant hemisphere. The sustained component of placebo analgesia involves emotional areas, located in temporal and parahippocampal cortices [21]. 

The activation of dopaminergic system when giving placebo has been documented by using PET with the D2/D3 receptor–labeling radiotracer 11Craclopride. One study performed on healthy subjects proved that intravenous placebo induced dopamine release at the basal ganglia level [22]. In patients with Parkinson's disease, placebo administration produces dopamine release in both dorsal and ventral striatum, as well as in the limbic system. By providing dopamine at the dorsal striatum level, the alleviation of motor symptoms in Parkinson's disease appears as a logical consequence. But the activation of ventral striatum and nucleus accumbens denotes the implication of the dopamine–mediated reward system, mechanism that might be responsible for part of the placebo effects not only in Parkinson's disease, but also in other medical disorders [23,24,25]. The degree of dopaminergic activation seems very important at the level of nucleus accumbens, this being correlated with a subject's expectation of analgesia, actualization of this expectancy during the trial and amplitude of analgesia [16]. The dopaminergic system is thought to be the mediator involved in the placebo effect on neuroimmunomodulation as well [26]. 

The serotoninergic system seems involved in the placebo effects observed in depressive patients, as one study that used PET to compare the changes in brain glucose metabolism induced by placebo versus fluoxetine, showed. During placebo administration, the metabolic rate was increased in the prefrontal, anterior and posterior cingulate, premotor, posterior insula, parietal cortex and decreased in subgenual cingulate, parahippocampus and thalamus; similar changes were found during fluoxetine treatment. However, changes in additional subcortical and limbic areas were seen only with fluoxetine, and these are thought to be responsible for the sustainability of treatment effects [27].

One study investigated the placebo effects on EEG recordings, and found that placebo induced a decreased P2 amplitude and an increased N2 amplitude, with the focus in the proximity of the posterior cingulate. Several studies have shown that P2 and N2 are linked to visual emotional stimuli, so their variation in amplitude corresponds to an emotional mechanism of the placebo effect [28]. 

The placebo effect has also been studied in animals; for instance, one study performed in mice offered proofs of the existence of opioid, naloxone reversible, and non-opioid, naloxone non–reversible, components of placebo analgesia [29].

### Neurological disorders and placebo effects Parkinson's disease

Clinical trials investigating antiparkinsonian drugs, deep brain stimulation or transplantation of fetal dopaminergic neurons versus placebo, revealed a significant improvement of motor function in the placebo arms. When a strict assessment of placebo–associated improvement was undertaken, by using the Unified Parkinson's Disease Rating Scale (UPDRS) motor scale the placebo response rates (improvement in the UPDRS motor score of at least 50% or reduction by at least two points on two different UPDRS items at one visit), were found to be of approximately 16% across multiple studies. The placebo response was more pronounced in patients with higher UPDRS scores at baseline, the disease form with motor fluctuations and when more invasive procedures were undertaken. The observed improvements were distributed similarly during a 6–month period [30].

The expectation of improvement triggered by the administration of placebo produces the release of dopamine in the striatum, as shown by positron emission tomography using raclopride, and this supplemental dopamine leads to the alleviation of motor symptoms in Parkinson's disease [25,31]. 

It was questioned in one study if the price of parkinsonism improvement when using placebo is the worsening of dyskinesia, as dyskinesia itself is considered to be generated by an excess of dopamine. However, the results contradicted this hypothesis: fewer patients showed placebo–related dyskinesia exacerbation compared with the alleviation and there was a lack of correlation between the changes in parkinsonism and changes in dyskinesia. These results were explained by the complexity of mechanisms underlying dyskinesia, as it is not a simple hyperdopaminergic state, but it is also due to receptor sensitization and pulsatile activity of dopamine. Moreover, other neurochemical mechanisms are thought to mediate the improvements of dyskinesia seen with placebo administration, such as glutamatergic, GABAergic, alpha_2_ adrenergic, serotonergic 5HT_1A_ and 5HT_2A_, opioid, histamine H_3_, adenosine A_2A_ receptors, the monoamine transport and cannabinoid CB1 receptors systems. The placebo effects in both parkinsonism and dyskinesia seem to be mediated by the alteration of NMDA receptor complex, which is involved in the expectation and reward mechanisms [32]

One study investigated the quality of life (QoL) during one year of double–blind follow–up in patients with advanced Parkinson’s disease who received either transplantation of human embryonic dopamine neurons or sham surgery. QoL was assessed as a composite index of several scales commonly used in Parkinson's disease, including Unified Parkinson's Disease Rating Scale (UPDRS) and Parkinson's Disease Stress Scale, as well as more general scales that evaluated the physical, emotional and social functioning. It was discovered that both groups had a statistically significant improvement in physical functioning over the one–year period of follow–up, with no significant differences in the overall QoL in the initial phase of the study.  This study revealed a strong placebo response in Parkinson's disease, thought to be elicited by the extreme nature of the placebo used (brain surgery) and by the fact that no clues regarding the actual treatment could be perceived neither by the patients or the medical staff during the follow–up period [33]. The placebo effect has also been investigated in parkinsonian patients who had been implanted electrodes for deep brain stimulation (DBS); the hand movement was faster when patients received a positive influence with the use of a placebo procedure [34]. Another study investigated the activity of single neurons in the subthalamic nucleus in patients who had been implanted with electrodes for DBS; the placebo–responders displayed a significant decrease of neuronal firing in the subthalamic nucleus during clinical improvement, which was reported by both the patient and the doctor [11].

The demographic information regarding the placebo effect in Parkinson's disease was obtained by an extensive search of the articles on this topic, published from 1969 to 1996 and listed in the Parkinson Study Group database for Deprenyl and Tocopherol Antioxidative Therapy of Parkinsonism (DATATOP). The survey concluded that the placebo effect did occur in Parkinson's disease, and it was not influenced by age, sex, religion, educational level or duration of the disease [35].

#### Neuropathic pain

A review of fourteen studies that investigated gabapentin versus placebo in various types of neuropathic pain (post–herpetic neuralgia, diabetic neuropathy,  cancer related neuropathic pain, phantom limb pain, Guillain Barre syndrome, spinal cord injury pain) reported an average placebo response of 19% [36].

The placebo effect in neuropathic pain seems influenced by the intensity of pain at baseline and by the site characteristics, reported an analysis of three clinical trials that investigated lamotrigine versus placebo. The amplitude of placebo effect in reducing pain (on Pain Intensity Numerical Rating Scale) was greater for patients with high baseline pain scores and for patients belonging to sites with a faster recruitment rate [37].

Some studies reported a decrease in the function of opioid and dopaminergic systems in patients with chronic pain syndromes, which suggested a diminished neurobiological availability to respond to placebo treatments [16].

#### Headache

One investigation that included 13 placebo–controlled trials performed in children and teenagers with acute migraine, found a rate of pain relief ranging between 38% and 53% (a pooled placebo response of 46%), and a rate of pain–free at 2 hours varying between 17% and 26% (a pooled placebo response of 21%). These rates were inferior to those observed in North American clinical trials, leading to the conclusion of a wide variability of the placebo effect in pediatric migraine [38].

A recent synthesis of the results of several placebo–controlled clinical trials run in children and adolescents with migraine reported the following placebo response rates: 27.1% for pain free after 2 hours and 56.9% for pain relief after 2 hours, in the parallel group trials [39].

A review of 11 clinical studies that investigated active treatments versus placebo in acute migraine revealed a wide range of placebo response rates, between 7–50% for pain decrease and 7–17% for pain cessation after 2 hours. The pooled placebo response rate was of 30% across these studies, with variations above and below this rate according to the primary endpoint, patient characteristics and design of the study [40].

In a systematic review of 11 placebo–controlled trials that investigated the treatment of acute migraine with analgesic drugs, an alleviation of pain was obtained in approximately 30% and pain cessation after 2h in 9% of the patients treated with placebo. One meta–analysis that included studies, which compared triptans with placebo, revealed an average rate of 30% placebo responders when the endpoint was pain relief and, 4–9% when the endpoint was the pain–free state. Another meta–analysis performed on 98 studies on migraine attacks gave a rate of 28.6% of patients who had pain improvement after 2 h, and 8.8% of patients who became free of pain after receiving placebo. The placebo response is lower in migraine prophylaxis: one meta–analysis, which included trials that compared propranolol to placebo, 14.3% of patients succeeded in attaining a reduction of > 50% in migraine frequency. A better response rate emerged from a meta–analysis of 32 studies that investigated migraine prophylaxis–the pooled placebo–response rate was of 21% [41].

The rates of placebo response reported in clinical trials that investigated migraine were approximately equal to those observed in clinical trials performed in cluster headache patients. A review of six randomized, placebo–controlled studies, which investigated acute cluster headache, showed a rate of placebo responders ranging between 7% and 42%, for the endpoint represented by complete remission or mild headache. As for the prophylaxis of cluster headache, placebo was found to be effective in 14% to 43% of patients, based on the results of other two clinical trials [42].

#### Multiple sclerosis

In multiple sclerosis clinical trials it is particularly difficult to separate the placebo effect from the natural history of the disease, as many cases have an unpredictable remission–relapsing pattern. Even if the exact cause of the phenomenon has not been established, several studies investigating interferon beta–1a proved that a decrease in the number of lesions seen on the MRI scan, also occurred in the placebo groups [6].

#### Epilepsy

A recently published meta–analysis, which included 54 studies, that investigated antiepileptic drugs versus placebo in more than 11 000 adults and children with refractory epilepsy, emphasized the small difference in effectiveness between antiepileptic drugs and placebo. The weighted pooled–risk difference for a 50% decrease of seizure frequency in the whole sample of adults and children was of 6% and 21%, respectively. In the same publication, a parallelism was made with the results of a previous meta–analysis on the same topic, which gave a rate of 0–2% for placebo in seizure–free patients [43].

An overview included a total of 28 clinical studies that investigated the effectiveness of several antiepileptic drugs (gabapentin, lamotrigine, tiagabine, topiramate, vigabatrin, and zonisamide) versus placebo as a treatment of refractory partial epilepsy. In the placebo arms, a response (defined as a reduction of seizure frequency of at least 50%) was obtained in 0–18% of the patients [44].

A recent meta–analysis that included 27 clinical trials which investigated antiepileptic drugs versus placebo in adults with focal drug–resistant epilepsy showed a pooled placebo response of 12.5% [45]. 

Another meta–analysis that included 32 studies about drug–resistant partial epilepsy (investigating gabapentin, levetiracetam, lamotrigine, oxacarbazepine and topiramate versus placebo) concluded that the average placebo response rates were of 19% in children and 9.9% in adults. This responder rate, which was 1.9 times higher in children than in adults, was stable across all antiepileptic drug trials. The reason for a better response to placebo in children is unclear; it may be due to a placebo effect by proxy, as the parents are those who report the outcome. However, since the studies did not include non–interventional groups, it cannot be estimated how much of this effect is due to other factors besides placebo, like Hawthorne effect, regression to the mean or the natural history of the disease [46].

One review of meta–analyses of randomized, placebo–controlled trials of antiepileptic drugs versus placebo included in Cochrane Library led to the conclusion that 9.3–16.6% of patients in the placebo arm had a >50% reduction in seizure frequency. It has been estimated that this effect accounts for 20–50% of the effect produced by the active medications, indicating the need for a better delineation of the true efficacy of each antiepileptic drug [47].

### Issues raised in randomized clinical trials (RCTs) and ethical aspects of using placebo

In RCTs, the real biological effectiveness is estimated as being the difference between the benefits observed in the active substance group and the placebo group; this might be a very reductionist approach, as the outcome may fluctuate with a variety of factors, including the details given during the informed consent process. When patients are told to participate in a placebo–controlled RCT, the chance of dropout due to ineffectiveness is bigger than in a comparison–controlled RCT, for the group that receives the same active medication. 

In order to isolate the true placebo effects in a clinical trial, some factors that may influence the outcome should be excluded: subject biases, ‘halo’ effect, Hawthorne effect and natural history of the disease. The first confounder can be controlled by blinding the subjects although this method is not invulnerable, because the adverse event profile might provide a clue regarding the true administered treatment [48]. However, the last factor remains a serious issue, as nowadays quite a few clinical trials have a non–treatment group to which the placebo group could be compared [6,16]. 

Given the fact that the placebo effect is responsible for the variability of the results in RCTs , several methods have been proposed to minimize it. One of them is the exclusion the placebo responders during wash-in periods; this approach is somehow unethical, because it refuses the chance of active treatment to a whole category of patients, and raises questions about the generalization of the study results [6] . 

In order to scrutinize the patients' opinion about receiving placebo in a randomized controlled trial, a qualitative sub–study that consisted of using the patient questionnaires was incorporated in a larger RCT. At the end of the RCT, the answers provided by the patients who received placebo were analyzed and the following conclusions were drawn: the fear of receiving placebo instead of active treatment was constant; they did not describe their feelings as ‘expectation’, but ‘hope’ of improvement; the vast majority reported a degree of improvement, psychosocial or/and symptomatic; there was persistent concern that the improvement might have been due to the normal evolution of disease or placebo effects [49].

One survey performed by giving questionnaires to patients of primary care clinics showed that 59% of the patients are open to the idea of participating in a placebo–controlled trial, in order to support the development of a new treatment and help other patients. The patients' understanding of the placebo effects was generally poor, with a tendency of underestimating its effectiveness [50]. 

Ethical concerns have been raised regarding the use of deception in neuroscience, as being a common approach thought to secure the scientific validity of the results; more than that, there is a lack of transparency in the published papers about the use of deception. Even if a written informed consent is formally obtained, the subjects are not actually informed about either the placebo use, or the purpose of the research. The U.S. federal regulations allow ‘a consent procedure which does not include, or which alters, some or all of the elements of informed consent...or waives the requirement to obtain informed consent’, when the risks for the patients are minimal and their welfare is not compromised, the research could not be performed otherwise and the debriefing procedure is used after the study [51].

The use of placebo in clinical trials is ethically acceptable only when the risks of not getting the active treatment are not very high for the patients. In order to balance the scientific and ethical needs and make the appropriate decisions, periodical reassessments of every particular disease, with its subtypes and stages, are undertaken by expert committees. For instance, an international committee has recently evaluated the ethics of running placebo–controlled clinical trials in relapsing multiple sclerosis; it was decided that the trials were still ethically acceptable, only if the active treatments were refused, inefficient or unavailable [52].

The use of sham surgery for neurological disorders has also been debated, as a necessary approach for the establishment of  the scientific validity of new surgery procedures in clinical trials, but respecting the safety and well–being of the patients. In order to address the ethical requirements, several conditions for the inclusion of  patients in this type of trials, have been proposed: the goal of the study is to bring new information that is scientifically valuable, there are no solid alternatives to using placebo, the risk of placebo is below an acceptable research risk, and, the patients authorize the use of deception to blind the placebo arm [53].

## Conclusions

Since 1955, when Beecher reported a rate of approximately 30% of placebo–responders in studies on analgesic drugs, further steps have been taken in understanding the placebo effect. More experience earned with the placebo effect in clinical trials enabled individualization of its impact on different pathological conditions, and a wide range of placebo–response rates have been acknowledged.

One published article gives a range of 5–65% for the placebo–induced improvements, the rate varying according to the particular CNS disease and sample size investigated in that study [16].

Given the fact that testing in a randomized placebo–controlled trial represents a crucial stage in the development of a new drug, in almost all therapeutic areas, a better understanding of the placebo effect has become of uttermost importance nowadays. The general current recommendation is to minimize the placebo effect in clinical trials, and the simplest way of doing that is to avoid making suggestions to patients. Studying the placebo effect in neurological disorders might provide better clues to a deeper understanding of the mechanisms involved, as it has been demonstrated by using performant–imaging techniques in the last several years. The applicability of this research area can be extremely vast, not only in neurology, but also virtually, in all medical fields.

The active drugs have been always preferred as a more potent and reliable therapy of a disease, however, their success is not absolutely guaranteed. More than that, there are still neurological disorders with limited treatment options. Using placebo in these cases, as a last resort, might represent a humanitarian decision, and should be tried. In general, the clinicians' tendency is to recommend enhancing placebo effect in common medical practice, as a benign and potentially effective part of the therapeutic process.
